# A multidimensional database of in-game player movements (Actions and events) in gaelic football

**DOI:** 10.1016/j.dib.2024.111082

**Published:** 2024-10-28

**Authors:** Valerio Antonini, Dermot Sheridan, Mark Roantree

**Affiliations:** a School of Computing, Dublin City University, Dublin, Ireland; b Insight Centre for Data Analytics, Dublin, Ireland

**Keywords:** Wearable devices, Sensor data, Sport analytics, Multidimensional analysis

## Abstract

Research in field sports often measures the performance of players during competitive games with individual and time-based descriptive statistics. Data is generated using GPS technologies, capturing simple data such as time (seconds) and position (latitude and longitude). While the data capture is highly granular and in relatively high volumes, the raw data are unsuited to any form of analysis or machine learning functions. The dataset presented here is created through a data engineering process, driven by domain experts, to transform the GPS coordinates into a series of (player) actions. Using 14 outfield players from each of 11 games, we present a database comprising 12 variables and almost 160k actions. Its reuse potential is targeted at machine learning researchers, sport scientists and coaches who may have different requirements represented as different analytical queries. This dataset is dimensional in nature, facilitating a rich set of analytics across dimensions such as game, player, action type and duration.

Specifications Table

**Table:** 

Subject	Sport Science
Specific subject area	*Sport analytics: player movement and effort during competitive games analyzed in terms of speed, speed variation, duration, and distance covered.*
Data format	Processed from raw GPS to Actions. Data provided in CSV and MySQL database dump.
Type of data	Comma separated .csv file (dataset with numbers and labels). .SQL database compatible with MySQL.
Data collection	Data were collected using a micro-GPS sensor device (STATSports Apex 10 Hz, N. Ireland), placed on each player's back, during 11 competitive Gaelic Football games during the years 2019–2020–2021. The sensor provides for each second, 10 consecutive values for the following variables: latitude, longitude, and speed (m s^-1^). The speed numerical values are converted to actions labels (‘Walking’, ‘Jogging’, etc.) by applying velocity thresholds known in sport literature. Consecutive rows sharing the same action label are aggregated together.
Data source location	*Region: Europe* *Country: Ireland*
Data accessibility	Repository name: Zenodo Data identification number: 10.5281/zenodo.13144848. Direct URL to data: https://zenodo.org/records/13144849
Related research article	Antonini, V., Mileo, A., & Roantree, M. (2024). Engineering Features from Raw Sensor Data to Analyse Player Movements during Competition. Sensors, 24(4), 1308. https://doi.org/10.3390/s24041308. [1]

## 1 Value of the Data

• While GPS data acquisition is pervasive in sport, there remains no available datasets describing movement action of players during official games. This data represents a unique opportunity to analyze player movements, change in speeds, and frequencies of accelerations during competitive Gaelic Football games, through the application of supervised and unsupervised machine learning tasks.
• The data can be reused by any researcher interested in analyzing players’ running performance, movements and structure of a team during official games. It provides insightful information about players’ behavior, change of speed over time and network structure of a team (centrality of players and actions and formed communities). It can also be used for different forms of cluster analysis, such as the identification of similarities or anomalies in sequences of actions, events, or players with similar levels of activity. Researchers can also use this data for time series research, as the data is a sequence of timed actions during game time.
• The dataset can be used for statistical analysis (descriptive statistics, correlation analysis, etc.) and for supervised or unsupervised machine learning tasks (prediction of future players speed and sequence of actions, analysis of changes in players’ distances, and clustering of similar movements or behaviors).
• Educators can use the dataset for project work involving data mining or problem-based learning with data at the core of the problem. It is also a practical database, usable for teaching SQL programming and the creation of data cubes.
• The data can be used by coaching professionals working in multiple field sports to quantify and validate players’ running performance or player load during game time.

## 2 Background

The application of machine learning to GPS data in sport analytics is still in an embryonic stage. There remain many sports yet to exploit this technology, including invasion sports in either individual, team, or tactical performance measurements [2]. Nevertheless, there is a growing interest in developing machine learning to predict player injury, fatigue, distances covered and patterns of movement. The data presented and described in this paper are created using the framework presented in [1]. That research presented a methodology to convert time series of GPS data (in terms of latitude, longitude, speed, and acceleration) to a set of features describing *action* movements performed by players during competitive games. Here, we present descriptive metadata, and in addition, explains how to form subsets or *data cubes* from the database in order to facilitate many different forms of analyses.

## 3 Data Description

This article describes the dataset of the actions performed by Gaelic Football players involved in 11 official inter-county games across the years 2019 to 2021. While the feature engineering process which generates the action dataset is described in detail elsewhere [1], a more detailed description of the dataset is provided here. Our method is based on the concepts of *actions* and *events*, concepts which are suited to rich forms of analysis and a broad range of machine learning functions. For each second of the game, speeds were converted to one of six *action* labels: ‘standing’, ‘walking’, ‘jogging’, ‘running’, ‘high-intensity running’, and ‘sprinting’, according to velocity thresholds widely accepted and defined in the literature [3]. At any point in time, each player is regarded as performing one of six possible actions. Players are regarded as being relatively static before commencing into some form of movement or sequence of actions. Thus, an *event* is a collection of sequential actions, bookended by either ‘standing’ or ‘walking’ actions.

The Actions dataset [4] (gaa_actions.csv) consists of 159,610 actions, each of them associated with an anonymized player, game identification number, and the Event to which they belong. As the online repository also provides a SQL dump [4] (gaa_actions.sql) and sample queries [4] (Queries.txt), this enables us to provide additional detail in the descriptions. Table 1 shows the columns of the Actions dataset. The *Action Counts* column provides an aggregation (count) for individual variables where possible.

**Table 1:** Actions dataset: features and descriptions.

Feature	Description	Values	Action Counts
GameID	Identifier for each game.	Random integer (11 distinct values) in the range 788–997.	788 14,260 811 14,480 838 13,033 869 14,899 873 14,784 889 14,155 893 14,864 934 13,617 946 14,925 973 14,526 997 16,067
PlayerID	Identifier for each player.	Random integer (35 distinct values in the range 114–331. Statistical data shows top 3 and bottom 3 values.	208 306 114 308 183 333 152 11,519 201 12,135 146 12,139
Half	Game Half	Integer: either 1 or 2	
Action	Action performed by the player.	‘standing’, ‘walking’, ‘jogging’, ‘running’, high-intensity running’, ‘sprinting’	Sprinting 1625 High Intensity Running 8629 Standing 19,124 Running 25,256 Jogging 51,868 Walking 53,108
EventID	EventID must be unique for each player/game combination.	Integer	
ActionID	Within each Event, the ActionID must be unique.	Integer	
Start_Time	Action start	Time	
End_Time	Action end	Time	
Start_Second	Action start (in seconds)	Integer	
Start_End	Action end (in seconds)	Integer	
Duration	Duration of the action in seconds (Start_End - Start_Second)	Integer	
Distance	Distance in meters covered during the action	Float	

Each action performed by players represents a row of the dataset whereas each feature represents a column [4] (gaa_actions.csv). This set of features enable the analysis of the actions performed by players. In Table 2, the correlation matrix for each feature is shown. Features are not correlated, with the exception of *EventID* and *Start_Second*. This information is important when using the dataset in machine learning algorithms.

**Table 2:** Correlation matrix of the numerical features.

	GameID	PlayerID	Half	ActionID	EventID	Start_Sec	End_Sec	Duration	Distance
GameID	1	0.05	0	0	0.04	−0.01	−0.01	−0.01	−0.01
PlayerID	0.05	1	−0.02	0.05	−0.03	−0.03	−0.03	0	−0.03
Half	0	−0.02	1	0.03	0.68	0.86	0.86	0.01	0
ActionID	0	0.05	0.03	1	−0.05	0.04	0.04	−0.02	−0.1
EventID	0.04	−0.03	0.68	−0.05	1	0.76	0.76	0.01	0
Start_Sec	−0.01	−0.03	0.86	0.04	0.76	1	1	0.02	0
End_Sec	−0.01	−0.03	0.86	0.04	0.76	1	1	0.02	0
Duration	−0.01	0	0.01	−0.02	0.01	0.02	0.02	1	0.74
Distance	−0.01	−0.03	0	−0.1	0	0	0	0.74	1

### 3.1 Dimension data: cubes

**Fig. 1:**
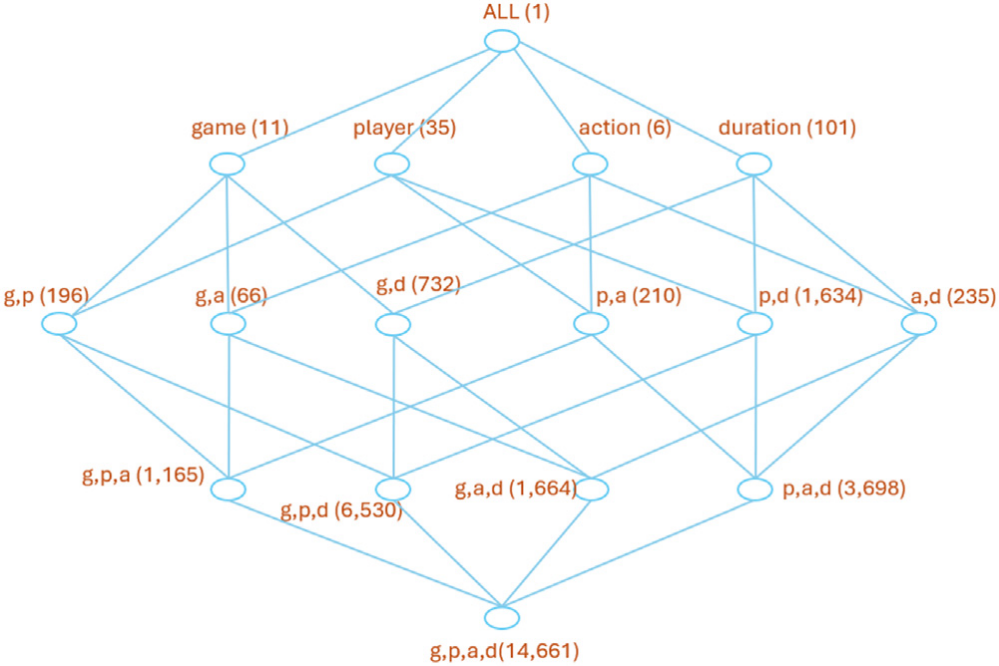
Lattice showing the 1, 2, 3 and 4 dimensional cubes with the counts for individual cuboids.

The dimensional nature of the dataset is illustrated using the lattice structure shown in Fig. 1, where levels from top (ALL) to bottom (game, player, action, duration) represent 0-D, 1-D, 2-D, 3-D and 4-D cubes respectively. In data warehousing terminology, each node in the lattice latest represents a cube and each cube comprises *n* cuboids [5]. In Fig. 1, the counts in brackets are for non-empty cuboids only. In other words, every game and player has at least 1 action as there are a total of 11 games and 35 players. However, not every game/player combination has actions (meaning that not all players played in every game) as this would imply a count of 35 × 11=385 *(g,p)* cuboids whereas in reality, only 196 cuboids exist.

Fig. 1 also illustrates how the overall dataset contains natural data subsets, each of which can be used for more fine-grained analyses. In effect, there are: 153 1-D datasets; 3073 2-D datasets; 13,057 3-D datasets; and 14,661 4-D datasets; all of which are regarded as data cubes. Obviously, as the overall dataset is shared across larger numbers of (higher dimensional) cubes, the action count for each cube is smaller.

**Fig. 2:**
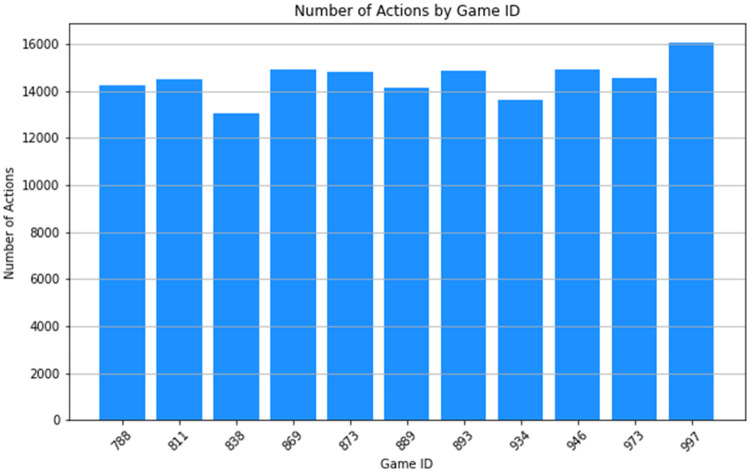
Bar chart display of the 1-D Game Cube, with 11 cuboids containing counts for all actions within each game. Query expression: “select GameID, count(*) from action_dataset group by GameID.Cube”.

In Fig. 2, the 11 game cuboids together with their (action) counts, are shown. The number of actions occurring in each game is broadly similar, although it is clear that game 838 has the least number of actions while game 997 has the most actions.

**Fig. 3:**
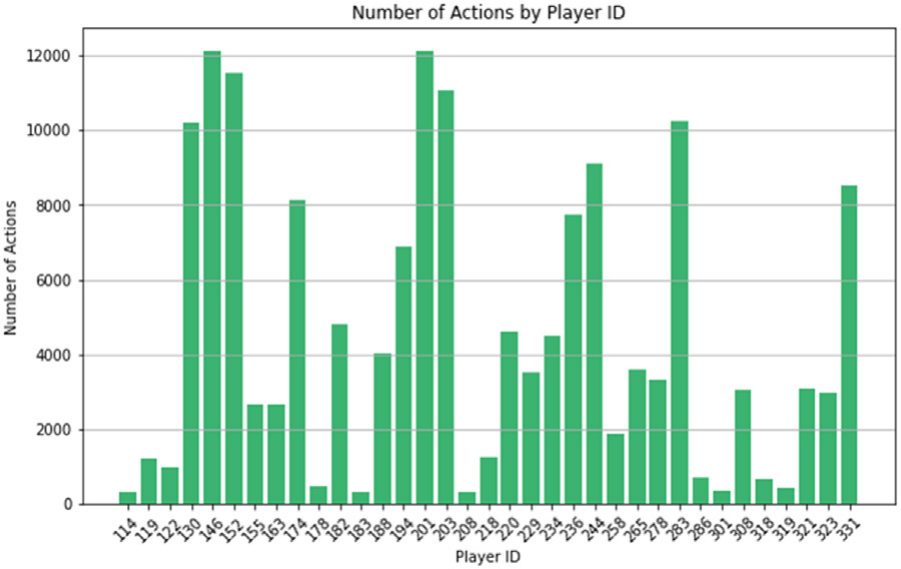
Bar chart display of the 1-D Player Cube with 35 cuboids containing counts for actions by player. Query expression: “select PlayerID, count(*) from action_dataset group by PlayerID”.

In Fig. 3, a similar 1-D illustration displays the action count per player. This graph highlights players who played a high number of games (or minutes) and those who played very little.

**Fig. 4:**
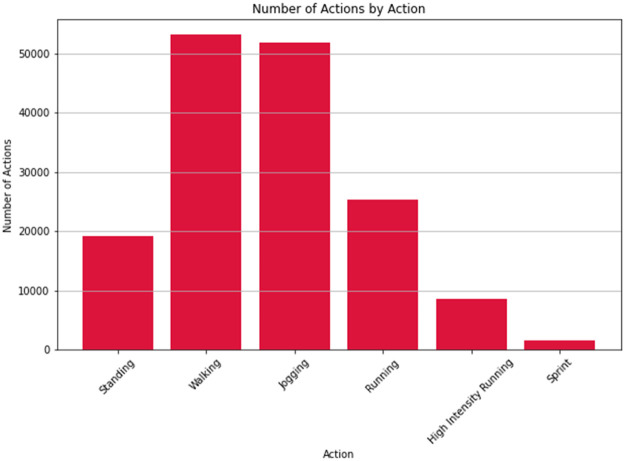
Bar chart display of the 1-D Action Cube containing 6 cuboids. Query expression: “select action, count(*) from action_dataset group by action”.

**Fig. 5:**
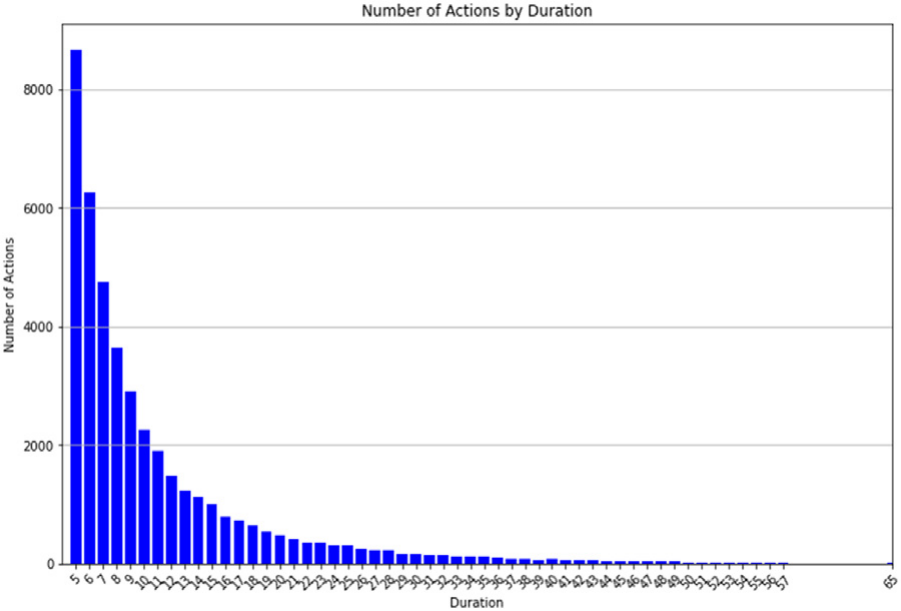
Bar chart display of the 1-D Duration Cube filtered to create only 54 cuboids, as per Example 1.

Fig. 4, Fig. 5 display the remaining 1-dimensional cubes. Fig. 4 illustrates those actions which are most common, while, as expected, high intensity actions are less common. Fig. 5 contains only 54 of the 101 cuboids in the database using the iceberg query [6] shown in *Example 1*. This type of query is useful in eliminating data cuboids containing very little information and provides a good example of how to create smaller, more focused data assets from the original large dataset.

**Example 1.**

SELECT duration, count(*) FROM action_dataset

where duration ≥ 5

group by duration

having count(*) > 10

order by count(*) desc

**Fig. 6:**
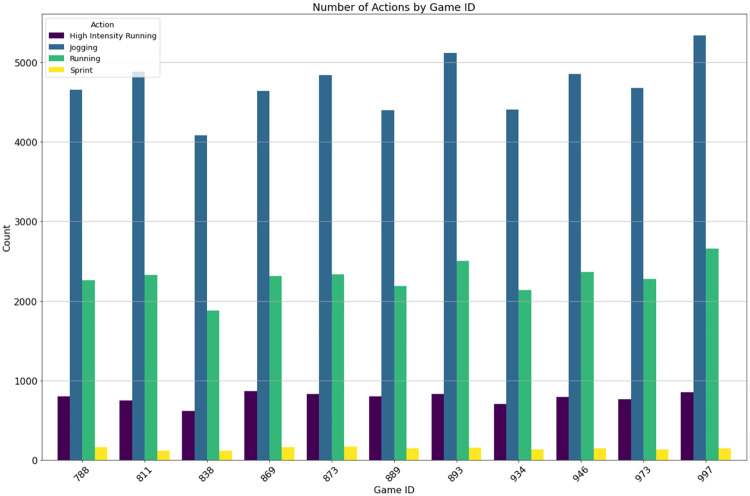
Bar chart display of the 2-D Game x Action Cube with standing and walking actions removed. Actions of <3 s are also removed.

The results in Fig. 5 show a clear decrease in duration count as the size (time interval) increases. Fig. 6 provides an example of a 2-dimensional (*Game x Action*) cube which is filtered to remove walking and standing actions. Example 2 illustrates the query which creates this data asset.

**Example 2.**

SELECT action, duration, count(*) FROM action_dataset

where duration ≥ 3 and action 〈 〉 ``standing'' and action 〈 〉

``walking'' group by action, duration

**Fig. 7:**
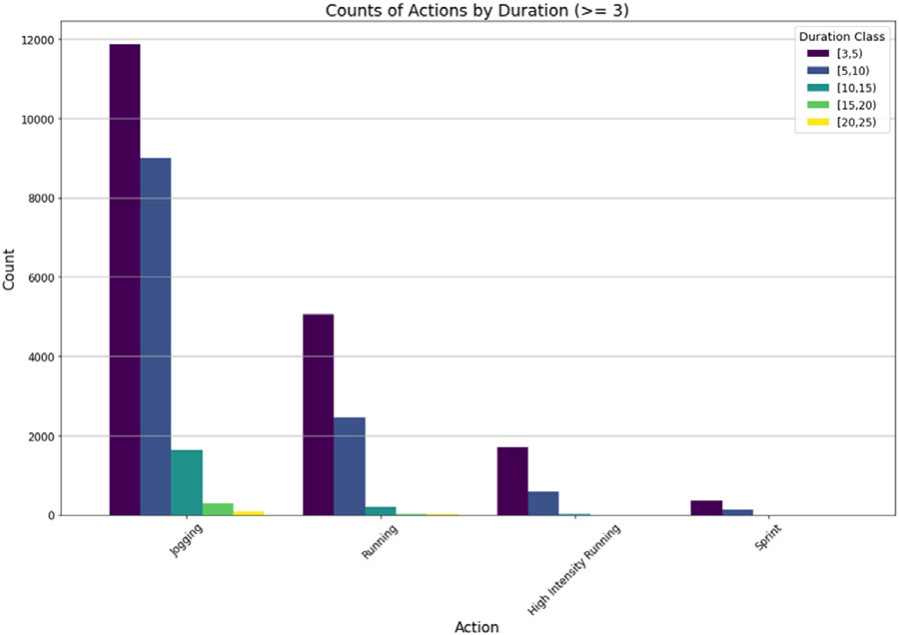
Bar chart display of the 4-D Hypercube.

The final cube is an example of a hypercube as it contains 4 dimensions. As the dimensional property of cubes increases, so does the number of cuboids. In general, the number of cuboids (distinct data assets) can be computed as Count(cuboids) = |d_1_| x |d_2_| x …x |d_n_| although some cuboids will be empty as shown in Fig. 1. The wider usage of these data cubes has already been demonstrated [7], where a graph database used a series of action cubes to analyze high-action areas, comparing games and players, to enable data-driven decisions by sports scientists. Fig. 7 illustrates the results of Example 2, presenting the counts of ‘jogging,’ ‘running,’ ‘high-intensity running,’ and ‘sprint’ actions over their respective durations.

## 4 Experimental Design, Materials and Methods

### 4.1 Data acquisition

Raw data were collected during 11 competitive Gaelic Football inter-county games throughout the seasons 2019–2020–2021. During game time, players were fitted with a micro-GPS sensor device (STATSports Apex 10 Hz), placed in a tight vest on their upper back. The 10 Hz STATSports Apex unit's validity and reliability has been assessed in previous research [8]. Other research supported these devices, reporting that the STATSports Apex 10 Hz unit had small error margins of around 1–2 % of the distances measured during the experiments [9]. This error was considered very low in relation to the significant volume in terms of distance and speed. For this reason, the research claims that the sensors can be confidently employed to measure distance variables during both training and match play [9].

The GPS unit records 10 observations of latitude, longitude, and speed (m s^-1^) for each second.

Latitude and longitude represent the geographical coordinates of a player's location on the field, captured 10 times per second. Latitude indicates the north-south position, while longitude reflects the east-west position. Together, they provide precise tracking of a player's movements in real-time. The player's instantaneous speed, measured in meters per second reflects the player's velocity at each point in time, enabling the calculation of movement intensity and player workload over the course of the match. Speed data are critical for evaluating sprint efforts, acceleration, and deceleration phases, contributing to performance and fatigue analysis.

The STATSports software makes the data available shortly after the end of the game. The software ensures that data are already cleaned and smoothed, and therefore, there is no need for any data pre-processing on the raw GPS data.. A sample of the raw data exported from the STATSports software is shown below in Table 3.

**Table 3:** Sample of raw data recorded by STATSports Apex 10 Hz sensor. The data shown have been made by the authors to resemble the original data.

PlayerID	Time	Latitude	Longitude	Speed (m s^-1^)
153	15:49:51.5	54.62311	−7.23798	5.60
153	15:49:51.6	54.99321	−7.23799	5.24
153	15:49:51.7	54.99327	−7.23788	5.01
153	15:49:51.8	54.99328	−7.23777	4.78

### 4.2 Data transformation

While a detailed description of the process to transform raw data into the action dataset was presented in [1], it is useful to provide a brief outline here. The initial step is the data aggregation step, which is necessary to reduce the granularity of the data. The raw data, sampled at 10 Hz (10 observations per second), are aggregated to 1-second intervals. This is achieved by averaging the GPS data within each 1-second window: the latitude and longitude are converted to the centroid of the positions during the second, and speed is averaged. Next, each speed value $x_{i}(i\;=1,\;2,..,\;n$), where i is a point in the game represented in seconds and n the final second of the game, is converted to an action using the labelled thresholds (Table 4). No standardized set of speed thresholds is available for invasion team sport to classify players’ speed into a describing label indicating the speed zone. For the purposes of this study, we adopted the speed thresholds suggested by [10], which are widely used in invasion team sport research.

**Table 4:** Speed thresholds and relative zone.

Speed (m s^-1^)	Speed Zone
0≤$x_{i}\;\leq 0.194$	Standing
$0.194\;<x_{i}\leq 2$	Walking
$2<x_{i}\leq 4$	Jogging
$4<x_{i}\leq 5.5$	Running
$5.5<x_{i}\leq 7$	High Intensity Running
$x_{1}>7$	Sprinting

Next, the data are further aggregated from 1-second intervals to actions, which represents consecutive seconds spent in the same speed zone. For each player, consecutive rows with the same speed zone are merged into a single action with new features created. This intermediate dataset has the following columns: ‘GameID’ (unique identifier for each game),: ‘PlayerID’ (unique identifier for each player), ‘Start Second’ (the second of game at which the action stars), ‘End Second’ (the second of game at which the action ends), ‘Action’ (speed zone label), ‘Duration’ (duration in seconds of speed maintained in the same speed zone), ‘Distance’ (distance in meters covered during the action).

**Fig. 8:**
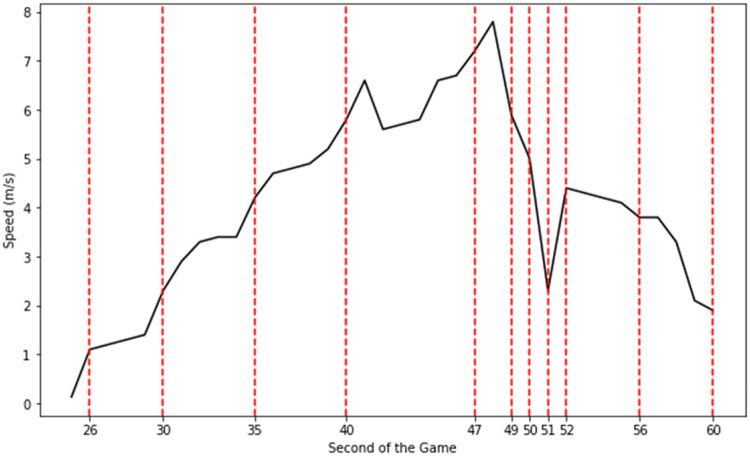
Visualization of an event from the speed time series. The event comprises a sequence of consecutive actions in which a player transitions through various speed zones before returning to a resting state. In this example, the event commences in the 25th second of the game and ends at 60th. It is composed of 12 actions as illustrated by the broken lines, starting with a 'standing' action, then the player performs some actions at higher speeds, and ends when he gets back to a resting state (in this case 'walking').

The last step is the application of the DetectEvent algorithm (presented in [1]) which is designed to identify sequences of actions, referred to as ``events'', from the action dataset. An event consists of a series of consecutive actions where a player moves through different speed zones before returning to a resting state, such as standing or walking (Fig. 8). Each event starts when the player transitions from a low-speed zone (standing or walking) to a higher-speed zone (e.g., jogging, running) and ends when the player returns to a resting state. The DetectEvent algorithm outputs a list of event IDs, where each unique event corresponds to a sequence of consecutive actions, starting and ending in a resting state. These events can vary in length and action composition, reflecting different phases of activity within the game. For example, an event might be a sequence of actions like:

• Walking → Jogging → Walking
• Walking → Jogging → Running → Walking
• Walking → Jogging → Running → High Intensity Running → Running → Jogging → Walking

By grouping such sequences into distinct events, the algorithm allows researchers to analyze periods of active movement within the game and understand player dynamics during those events.

A new column, 'EventID,' is added to the intermediate action dataset to assign a unique identifier to each event associated with the corresponding action. The resulting dataset forms the action dataset presented in this paper.

### 4.3 Data validation

To validate the action dataset and ensure it accurately reflects actual game dynamics the dataset was compared with existing literature on Gaelic Football (GF) to verify consistency in terms of distance covered and movement intensity.

The running profiles obtained from this dataset were compared to findings by [11], where 50 elite players were tracked using 4-Hz GPS units. In [11], an average distance of 8160 ± 1482 m (m) was covered, with 1731 ± 659 m at speeds ≥ 17 km/h and 445 ± 169 m at speeds ≥ 22 km/h. The current study shows comparable results: an average distance of 8633.8 ± 1573.6 m, 1453.6 ± 552.7 m at speeds ≥ 17 km/h, and 503.5 ± 205.1 m at speeds ≥ 22 km/h. Average speed and peak speed were also consistent with previous studies.

Contrary to [11], this study found no significant reduction in high-speed distance or sprinting distance between the first and second halves. However, reductions in distance covered were similar across quarters when compared to a separate study by [3], showing a decrease in distance from the 1st to the 2nd, 3rd, and 4th quarters, with statistically significant differences between the 1st and 4th quarters.

Similarly, [12] found declines in jogging and running distances between quarters. In this study, significant declines were detected only between the 1st and 4th quarters, aligning with those findings. Overall, the action dataset shows strong consistency with prior research, supporting the accuracy.

### 4.4 Data analysis

Excluding the goalkeeper, the average speed during the first half was measured at 2.84 ± 1.62 m s^-1^, while in the second half, it was 2.73 ± 1.61 m s^-1^. On average, there were 3945 ± 286 actions per game, with an average action duration of 2.9 ± 0.1 s, and the maximum duration averaged 25.2 ± 3 s. The analysis shows that the average number of actions per game decreases as speed increases, with more actions occurring at lower speeds: `jogging' had the most, followed by `running', `high-intensity running', and `sprinting'. A *t*-test revealed [2] a statistically significant difference in the mean duration of `high-intensity running' between the first half (2.0 ± 0.1 s) and the second half (2.2 ± 0.1 s), probably indicating fatigue. For other actions, the mean durations were 3.4 ± 0.1 s for `jogging', 2.3 ± 0.1 s for `running', and 2.1 ± 0.2 s for `sprinting'. Additionally, a statistically significant difference in the mean distance per `high-intensity running' action was observed between the halves, with 12.4 ± 8.7 m in the first half and 13.1 ± 9.6 m in the second half. Finally, the low intensity events `standing', `walking', and combinations of `standing' and `walking' were excluded from this analysis.

## Limitations

‘None’

## Ethics Statement

The study was conducted in accordance with the Declaration of Helsinki and approved by the Institutional Review Board (or Ethics Committee) of Dublin City University (protocol code DCUREC/2021/267 and date of approval 27 January 2022).

## CRediT Author Statement

Author Contributions: Conceptualization, V.A., M.R. and D.S.; methodology M.R.; software, V.A.; validation, V.A., D.S. and M.R.; formal analysis M.R.; writing–original draft preparation, V.A.; writing—review and editing, M.R.; visualization, V.A.; supervision, M.R. All authors have read and agreed to the published version of this manuscript.

## Data Availability

ZENODOA Database of In-Game Player Movements (Actions and Events) in Gaelic Football (Original data).
